# A bottom-up approach to estimating cost elements of REDD+ pilot projects in Tanzania

**DOI:** 10.1186/1750-0680-7-9

**Published:** 2012-08-09

**Authors:** Eduard Merger, Christian Held, Timm Tennigkeit, Tom Blomley

**Affiliations:** 1 UNIQUE forestry and land use, Schnewlinstrasse 10, 79098, Freiburg, Germany; 2 LTS International Ltd., Pentlands Science Park, Bush Loan, Penicuik, Nr. Edinburgh, EH26 0PL, Scotland, UK

**Keywords:** REDD+ economics, REDD+ cost elements, Opportunity costs, REDD+ implementation costs, Transaction costs, Institutional costs

## Abstract

**Background:**

Several previous global REDD+ cost studies have been conducted, demonstrating that payments for maintaining forest carbon stocks have significant potential to be a cost-effective mechanism for climate change mitigation. These studies have mostly followed highly aggregated top-down approaches without estimating the full range of REDD+ costs elements, thus underestimating the actual costs of REDD+. Based on three REDD+ pilot projects in Tanzania, representing an area of 327,825 ha, this study explicitly adopts a bottom-up approach to data assessment. By estimating opportunity, implementation, transaction and institutional costs of REDD+ we develop a practical and replicable methodological framework to consistently assess REDD+ cost elements.

**Results:**

Based on historical land use change patterns, current region-specific economic conditions and carbon stocks, project-specific opportunity costs ranged between US$ -7.8 and 28.8 tCO_xxxx_ for deforestation and forest degradation drivers such as agriculture, fuel wood production, unsustainable timber extraction and pasture expansion. The mean opportunity costs for the three projects ranged between US$ 10.1 – 12.5 tCO_2_. Implementation costs comprised between 89% and 95% of total project costs (excluding opportunity costs) ranging between US$ 4.5 - 12.2 tCO_2_ for a period of 30 years. Transaction costs for measurement, reporting, verification (MRV), and other carbon market related compliance costs comprised a minor share, between US$ 0.21 - 1.46 tCO_2_. Similarly, the institutional costs comprised around 1% of total REDD+ costs in a range of US$ 0.06 – 0.11 tCO_2_.

**Conclusions:**

The use of bottom-up approaches to estimate REDD+ economics by considering regional variations in economic conditions and carbon stocks has been shown to be an appropriate approach to provide policy and decision-makers robust economic information on REDD+. The assessment of opportunity costs is a crucial first step to provide information on the economic baseline situation of deforestation and forest degradation agents and on the economic incentives required to halt unsustainable land use. Since performance based REDD+ carbon payments decrease over time (as deforestation rates drop and for each saved ha of forest payments occur once), investments in REDD+ implementation have a crucial role in triggering sustainable land use systems by investing in the underlying assets and the generation of sustainable revenue streams to compensate for opportunity costs of land use change. With a potential increase in the land value due to effective REDD+ investments, expenditures in an enabling institutional environment for REDD+ policies are crucial to avoid higher deforestation pressure on natural forests.

## Background

In the process of developing national REDD+ frameworks, investors, donors and policymakers are highly interested in cost information in order to develop strategies, allocate budgets, and to assess the effectiveness of green house gases (GHG) emission reductions from avoiding deforestation and forest degradation, conservation, sustainable management of forests, and enhancement of forest carbon stocks in developing countries [1].

Several REDD+ cost studies have been conducted [2-6] asserting that payments for maintaining forest carbon stocks have significant potential to be a cost-effective climate change mitigation and human development measure. These studies mainly build upon top-down global empirical or global simulation models using highly aggregated data without taking into consideration regional differences. Moreover, these REDD+ cost studies have mainly focused on opportunity costs and partly on implementation costs without taking into account transaction and institutional costs, thus potentially underestimating the actual costs of REDD+ [7]. The results of global REDD+ cost estimates can only be used to a limited extent for many Sub-Saharan African countries, as most of the studies [1,2,5] were conducted in REDD+ countries (e.g. Brazil, Bolivia, Cameroon, Democratic Republic of the Congo, Ghana, Indonesia, Malaysia, and Papua New Guinea) with relatively high forest carbon stocks (i.e. > 100 tC/ha [8]) in the moist tropics. In contrast, many Sub-Saharan countries, such as Tanzania, host areas of tropical dry forest ecosystems with carbon densities of less than 50 tC/ha [9-11]. Moreover, Tanzanian markets are often not formalized and drivers of deforestation and forest degradation cannot be directly compared due to differing economic, socio-cultural, political and demographic factors [12,13]. This reinforces the importance of regional differentiation when assessing REDD+ economics.

In a previous study conducted in 2011, REDD+ opportunity and implementation costs for Tanzania were estimated using a bottom-up approach by taking into consideration regional variations at district-level [14]. Opportunity costs in 53 districts for avoiding charcoal and agricultural expansion ranged between US$ 1.90 – 13.40 tCO_2_ (median US$ 3.90 tCO_2_), indicating the high variability of opportunity costs within the country. Implementation costs for avoiding GHG emissions through investments in doubling agricultural output and in more efficient charcoal production exceeded opportunity costs, and ranged between US$ 1.63 – 17.05 tCO_2_ (median US$ 6.52 tCO_2_). It was concluded that the opportunity and implementation costs of avoiding GHG emission from REDD+ are likely to exceed estimates generated from previous global top-down models [14]. Building upon these findings, our work focuses on a practical bottom-up assessment of the full range of REDD+ costs, including opportunity, implementation, transaction and institutional costs for three Tanzanian REDD+ pilot projects (Jane Goodall Masito Ugalla Ecosystem Pilot Area (JGP); Tanzania Forest Conservation Group / Mjumita project (TFCG) - Kilosa site and Lindi site; Table 1). In our investigation, we assessed the opportunity costs of all existent drivers of deforestation and forest degradation at the project sites. We also take into account market structures and estimate the actual value of land to land users in the respective project regions. This approach provides a robust and practical REDD+ cost element assessment framework relevant also to other REDD+ pilot initiatives.

**Table 1:** Characteristics of selected REDD+ pilot projects

**Project title**	**Jane Goodall Masito Ugalla ecosystem pilot area**	**Tanzania Forest Conservation Group (TFCG) / Mjumita project - Kilosa site**	**Tanzania Forest Conservation Group (TFCG) / Mjumita project – Lindi site**
**Project location**	Mpanda and Kigoma districts	Kilosa district	Lindi rural district
**Total project area (ha)**	85,200 ha	148,825 ha	93,800 ha
**Major drivers of deforestation and forest degradation**	Unsustainable timber extraction and fuel wood collection; Shifting cultivation; Pasture (grazing cattle)	Unsustainable charcoal production; Shifting cultivation	Unsustainable charcoal production; Shifting cultivation
**Forest type**	Masito and Ugalla forests	Eastern Arc Forests	Coastal forest
**Project objectves / activities**	Conservation and alternative income generation	Conservation and alternative income generation	Conservation and alternative income generation
**Current status**	In the project description development phase	Project description for VCS finalised	In the project description development phase
**Annual deforestation rate over the past 10 years**	1.8%	0.35%	1.55%

## Results

### Land use classification and land use change patterns of REDD+ pilot projects

The land use classification in the pilot project areas is presented in Table 2. In the JGP (85,200 ha) we identified four major drivers of deforestation and forest degradation, including unsustainable timber and fuel wood extraction, agriculture and pasture expansion, each responsible for 21%, 28%, 37% and 14% of the total historical deforestation respectively over the past 10 years, equivalent to 14,163 ha (1.8 % annual deforestation rate). In the TFCG / Mjumita Kilosa project (148,825 ha) the annual deforestation rate was about 0.35 % resulting in deforestation of 9,208 ha. Out of this area, 46% had been caused by unsustainable charcoal production and 54% by agricultural expansion. In the TFCG / Mjumita Lindi project (93,800 ha), total deforestation amounted to about 16,808 ha with an annual deforestation rate of 1.55%. Unsustainable charcoal production and agricultural expansion were each responsible for 50% of the total deforestation.

**Table 2:** Mean carbon stock sof REDD+ pilot project land classification (tCO2/ha) (aboveground and belowground biomass)

**Land use type**	**Jane Goodall Masito Ugalla ecosystem pilot area**	**Tanzania forest Conservation Group (TFCG) / Mjumita project - Kilosa site**	**Tanzania forest Conservation Group (TFCG) / Mjumita project – Lindi site**
**Natural forest**	80.6	145.3	158.6
**Unsustainable timber extraction**	30.2		
**Unsustainable charcoal production**		16.1	53.8
**Unsustainable fuel wood production**	30.2		
**Agriculture (Shifting cultivation)**	15.4	16.1	53.8
**Pasture (Cattle grazing)**	22.2		

**Jane Goodall Masito Ugalla Project**: Accounting for aboveground and belowground biomass based on mean carbon stocks as measured in the Ground Forest Carbon assessment of the Masito Ugalla Ecosystem Pilot area [15]; **TFCG Mjumita – Kilosa:** Long-term average aboveground carbon stocks based on mean carbon stocks from 17 forest plots randomly distributed across the project area, derived by the project staff from local field surveys. Belowground carbon stocks are based on IPCC default root-to-shoot ratio for dry tropical forest [8]; **TFCG Mjumita – Lindi:** The mean carbon stocks are based on the average aboveground and belowground carbon stock from derived from local field surveys by TFCG Mjumita project staff. Belowground carbon stocks are based on IPCC default root-to-shoot ratio for dry tropical forest [8].

### Carbon stocks

All three projects plan to account, monitor and receive payments only for avoided deforestation from aboveground and belowground biomass carbon pools. Table 2 shows the carbon stock estimates for the identified land uses in the analysed REDD+ pilot projects. The mean natural forest carbon stocks in the JGP amount to 80.6 tCO_2_/ha, while in Kilosa and Lindi carbon stocks are almost twice as high at 145.3 tCO_2_/ha and 158.8 tCO_2_/ha respectively. With deforestation of one ha in the JGP, between 50 and 65 tCO_2_ are emitted, while in the TFCG Kilosa and Lindi projects the mitigation potential of one ha is significantly larger at 129 tCO_2_ and 105 tCO_2_, respectively.

### Economics of land uses

Following the methodological approach as described in the methods section, Table 3 presents the net present value (NPV) of each identified land use for the three analysed REDD+ pilot projects.

**Table 3:** Net present values for natural forest and drivers of deforestation and forest degradation over 30 years at a discount rate of 10% (US$/ha)

**Land use type**	**Jane Goodall Masito Ugalla ecosystem pilot area (JGP)**	**Tanzania forest Conservation Group (TFCG) / Mjumita project - Kilosa site**	**Tanzania forest Conservation Group (TFCG) / Mjumita project – Lindi site**
**Natural forest**	924	95	95
**Unsustainable timber extraction**	1,687		
**Unsustainable charcoal production**		1,662	1,290
**Unsustainable fuel wood production**	533		
**Agriculture (Shifting cultivation)**	2,806	1,232	1,023
**Pasture (Cattle grazing)**	1,348		

The NPV for natural forest in the JGP (U$ 924/ha) is significantly higher than in the TFCG / Mjumita Lindi and Kilosa projects (both US$ 95/ha). This difference is mainly due to the significantly larger use of non-timber-forest products and better marketing opportunities. In our modelling of unsustainable timber production for the JGP, which has led to deforestation and forest degradation over the past 10 years, we estimated that the forest is used for timber extraction over a period of seven years with an available extraction volume of 5.9 m³/year and a commercial recovery rate of 30%, sold at an average of US$ 112.5/m³ at the farm-gate. Subsequently the land is converted to agricultural land for shifting cultivation with four years of cultivation and 12 years of fallow period resulting in an NPV of US$ 1,687/ha. Unsustainable fuel wood collection, estimated assuming an annual average extraction rate of 3.6 tdm/ha over 30 years (annual increment 2.4 tdm/ha), results in a low NPV of US$ 533/ha mainly due to the low market prices (US$ 20/m³) for fuel wood in this region and the high level of household self-consumption, which is valued in the model. Agricultural land use in the JGP has a significantly greater NPV (US$ 2,806/ha) than in Kilosa (US$ 1,232/ha) and Lindi (US$ 1,023/ha) projects, mainly due to higher productivity of the land and its proximity to the border with the Democratic Republic of Congo, which provides good market access. Regarding unsustainable charcoal land use in the Kilosa and Lindi projects, we assumed that one ha under deforestation and degradation pressure is cleared for charcoal production within one year extracting about 28 tdm/ha. Assuming a mean charcoal kiln efficiency of 19% [16,17], this results in about 6.1 t of charcoal sold at a price of US$ 100/t at farm-gate. Afterwards, the land is used for shifting cultivation, mainly for sesame production, the most profitable and prevalent crop in Lindi (cultivation of two years and fallow for ten years) and beans in Kilosa (cultivation for five years and fallow for eight years). The resulting figures confirm the findings of Fisher et al [14] that charcoal and agriculture are economically the most profitable drivers of deforestation and forest degradation in Tanzania.

### REDD+ opportunity cost curve

**Figure 1:**
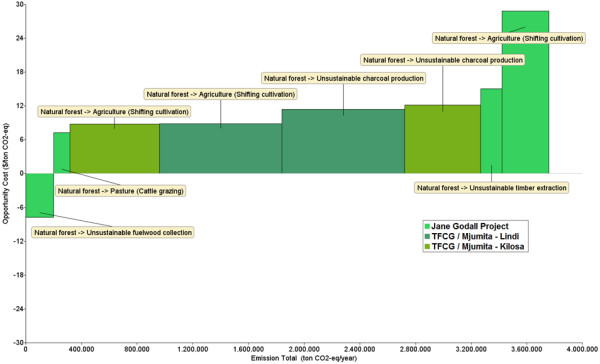
**REDD+ opportunity cost curve for three pilot projects in Tanzania (327,825 ha).** (Cost curve extracted from the Abacus visualisation tool).

Based on the aforementioned historical land use change patterns, carbon stock and economic data, the opportunity cost curve of avoiding the conversion of natural forest for the three pilot projects is presented in Figure 1. It is based on the historically deforested area of 40,179 ha that occurred on a total project area of 327,825 ha over the past 10 years. Figure 1 shows that the JGP has two relatively cost-effective options for reducing deforestation, i.e. avoiding unsustainable fuel wood collection and avoiding expansion of cattle grazing. Together, these two options have a total emission reduction potential of 318,489 tCO_2_ over 10 years (31,849 tCO_2_/yr), and the opportunity costs of land use change are negative (US$ -7.8 tCO_2_) for unsustainable fuel wood collection and US$ 7.3 tCO_2_ for avoiding the expansion of cattle grazing. The negative opportunity cost indicates that in the long-term, maintaining natural forest would result in a higher NPV than the NPV of unsustainable fuel wood collection and at the same time higher carbon stocks on a unit of land would be achieved in the long-term.

The two most expensive options are avoidance of unsustainable timber extraction and shifting cultivation. Together these two options have a total emission reduction potential of 488,791 tCO_2_ (48,879 tCO_2_/yr) at costs of US$ 15.1 tCO_2_ for unsustainable timber extraction and US$ 28.8 tCO_2_ for avoiding conversion undershifting cultivation. The Kilosa and the Lindi projects have a large mitigation potential from avoiding the expansion of shifting cultivation and unsustainable charcoal production. In the Kilosa project opportunity costs for shifting cultivation amount to US$ 8.8 tCO_2_, while for unsustainable charcoal production opportunity costs are US$ 12.1 tCO_2_. In total, avoiding conversion of natural forest to these land uses has the potential to reduce emissions by 1.19 MtCO_2_ over 10 years (118,923 tCO_2_/yr). Similarly, in the Lindi project the avoidance of land use change to shifting cultivation and unsustainable charcoal production incurs opportunity costs of US$ 8.9 tCO_2_ and US$ 11.4 tCO_2_ respectively, and could reduce emissions by 1.76 MtCO_2_ over a period of 10 years (176,181 tCO_2_/yr).

Taking the mean opportunity costs of each project, the JGP has opportunity costs of US$ 12.5 tCO_2_, while the TFCG / Mjumita projects have lower opportunity costs at US$ 10.1 tCO_2_ and US$ 10.3 tCO_2_ for the Lindi and Kilosa sites, respectively.

### Implementation, transaction and institutional costs

**Figure 2:**
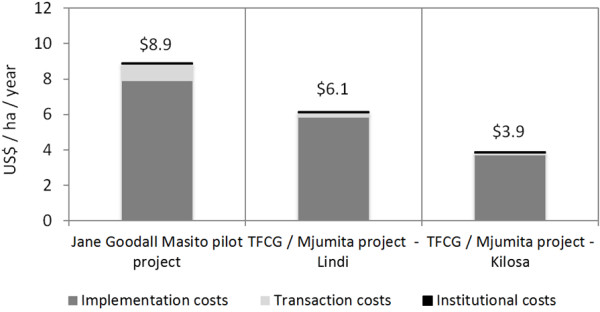
Implementation, transaction and institutional costs (US$/ha/yr).

For the quantification of the implementation and transaction costs of the REDD+ pilot projects, we used available REDD+ project budgets. In our estimates we assume that no leakage effects will occur and that opportunity costs will be compensated by the project interventions or performance based payments. Implementation costs comprised between 89% and 95% of total project costs (excluding opportunity costs), ranging between US$ 7.9 ha/year (JGP), US$ 5.8 ha/year Lindi project and US$ 3.7 ha/year in the case of the Kilosa project (Figure 2). Table 4 below summarises the budgeted implementation cost items of the projects.

**Table 4:** Budgeted implementation cost items of REDD+ pilot projects

**Project title**	**Budgeted interventions to reduce pressure on deforestation and forest degradation**
**Jane Goodall Masito Ugalla Ecosystem Pilot Area**	· Technical and management costs (administration, staff salaries, office operations and maintenance, equipment)
	· Training and capacity building of local communities and selected stakeholders relevant for REDD+ implementation
	· Formation of forest conservation civil society organizations (CBOs)
	· Training on sustainable forest management practices and development of forest management plans
	· Development and operation of participatory benefit sharing mechanisms
	· Training in business management and marketing
	· Project monitoring and evaluation
	· Consulting fees
**Tanzania Forest Conservation Group (TFCG) / Mjumita project - Kilosa site and Lindi site**	· Technical and management costs (administration, staff salaries, office operations and maintenance, equipment)
	· Review of forest management plans and support of participatory forest management
	· Training and capacity building of staff members and village trainers on REDD, participatory forest management and leakage prevention
	· Support to community communication processes and REDD+ awareness
	· Results-based payments to communities
	· Development and implementation of leakage avoidance measures and action plans (land use planning, agroforestry, tree planting among others)
	· Monitoring and evaluation
	· Participation in international

Transaction costs for measurement, reporting and verification (MRV) and other carbon market related compliance costs ranged between US$ 0.9 ha/year for the JGP, US$ 0.3 ha/year for TFCG / Mjumita – Lindi and US$ 0.2 ha/year for the TFCG / Mjumita – Kilosa project. These implementation and transaction costs will be covered by the project development entities.

In our study we assume that institutional costs are partly covered by the projects and partly by the government. One part of the institutional costs are comprised of a national average value of US$ 0.017 ha/yr, which is based on the budgets of the Forest Carbon Partnership Facility Readiness Preparation Proposal of Tanzania [18]. These costs are covered by the national government of Tanzania.

The other part of the institutional costs, to be covered by the pilot projects, are budget items related to institutional capacity building for district level governmental staff and knowledge dissemination of lessons learned which is an additional activity to build and strengthen REDD+ relevant institutions and capacity in Tanzania. The estimated institutional costs did not differ significantly among projects and amounted to US$ 0.07 ha/year for the JGP, US $0.07 ha/year for the TFCG / Mjumita - Lindi project and US $0.05 ha/year for TFCG Mjumita – Kilosa (Figure 2).

**Figure 3:**
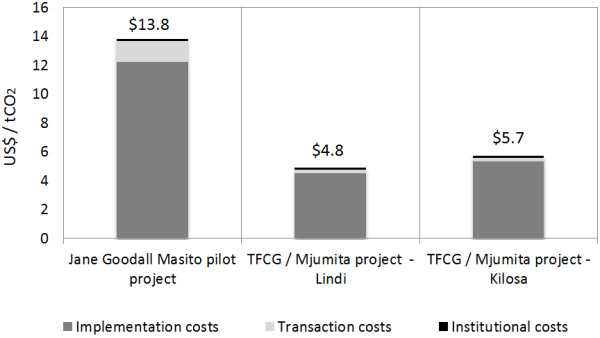
Total costs per avoided tCO2 emission (US$/tCO2).

On average, the JGP is expected to reduce 55,000 tCO_2_/yr (1.65 MtCO_2_ over 30 years) while avoiding deforestation on about 20,000 ha, which is equivalent to 68% of the project’s reference emission levels. For the TFCG / Mjumita Kilosa project, emissions reductions will average 102,000 tCO_2_/yr (3.06 MtCO_2_ over 30 years), equivalent to the avoided conversion of 23,700 ha, and an 86% reduction compared to the reference emissions level. In the Lindi project, the total emissions reductions are projected to be about 120,000 tCO_2_/yr on average (3.6 MtCO_2_ over 30 years) which is equivalent to avoided deforestation and forest degradation on 34,340 ha and 68% of the reference emissions level. Assuming that the entire project implementation will be paid by carbon payments, in the JGP the total cost of avoiding the release of one tCO_2_ would be US$ 13.8, while the TFCG / Mjumita projects cost between US$ 4.8 (Lindi) and US$ 5.7 (Kilosa) (Figure 3).

## Discussion

### REDD+ opportunity cost curve

In general there are three different approaches to estimate cost elements of REDD+, i.e. local empirical models, global empirical approaches and global simulation models [5,19]. The local empirical estimates are based on local surveys and information, estimating per-area costs ($/ha) and carbon density (tCO_2_/ha) of a particular REDD+ activity location. A review of 29 empirical studies identified opportunity costs of REDD+ in the range of US$ 0.84 – 4.18 tCO_2_ with a mean of US$ 2.51 tCO_2_[5]. Of these 29 studies, 28 had REDD+ costs at less than US$ 10 tCO_2_ with a mean of US$ 2.22 tCO_2_ for Africa. However, the studies quantified only opportunity costs and did not take into account other cost elements associated with implementation, measurement, reporting and verification, and institutional arrangements within REDD+ frameworks [1]. Global empirical models use local empirical data and combine these to produce global per-area costs of deforestation. These models use uniform estimates of carbon density (tCO_2_/ha) resulting in a global estimate of opportunity costs (US$/tCO_2_) [5]. This method was used for the Stern Review, undertaken by Grieg-Gran [20] who estimated global REDD+ costs in the range of US$ 2.76 – 8.28 tCO_2_ (midpoint: US$ 5.52 tCO_2_)_._ The study considered only opportunity costs and administration costs of REDD+ and used a mean forest carbon density of 390 tCO_2_/ha. This approach ignored the significant variations in carbon densities between regions and led to highly aggregated estimates with a large uncertainty level. Global simulation models such as Global Timber Model (GTM), Dynamic Integrated Model of Forestry and alternative Land Use (DIMA) and Generalized Comprehensive Mitigation Assessment Process Model (GCOMAP), estimate REDD+ costs by simulating the development of the world economy taking into account the forestry, agricultural and fossil fuel using energy sectors [6]. The result of simulations is to provide supply curves of REDD+ (price vs. quantity for emissions reductions in tCO_2_). These models mainly also focus on opportunity costs without taking into account other cost elements of REDD+. A published study has estimated the costs of reducing global deforestation by 50% by 2030 to be in the range of US$ 9.27 – 20.57 tCO_2_. Reducing African forestry related GHG emissions by half was estimated to cost between US$ 5.20 - 12.3 tCO_2_[6].

Opportunity costs are likely to vary significantly from place to place, depending on several factors such as agro-ecological conditions, climate, market access, scale of operation, inputs, technology and yields, so highly aggregated average values may be misleading [21,22].This is evidenced by the comparison of our opportunity costs estimates for three REDD+ pilot projects (mean project average of US$ 10.1 - 12.5 tCO_2_). Comparing these estimates with data from Tanzania [23] where opportunity cost estimates were based on maize and charcoal production, costs have been estimated to range between US$ 1.90 and 13.40 tCO_2_ with a median US$ 3.90 tCO_2_ for Tanzania.

As a basis for these estimates, IPCC [8] derived forest carbon densities in the range of 196 and 490 tCO_2_/ha (accounting for aboveground, belowground biomass, litter and soil carbon pools) were used. These are significantly higher than our reported project-specific carbon densities that range between 80.6 and 158.6 tCO_2_/ha (Table 2) and that account only for aboveground and belowground biomass. This explains the large difference in opportunity costs on a per tonne basis between the two approaches. Considering that most Tanzanian forests are classified as dry tropical forests under the IPCC classification where soil carbon stocks are normally over 100 tCO_2_/ha to a depth of 0–30 cm [8,24,25], accounting for the soil carbon pool could more than double potential carbon benefits when maintaining natural forests. Since monitoring for soil carbon can be very expensive [26], accounting for soil carbon would increase transaction costs as well, due to the larger investment and capacity required to include soil carbon in MRV systems. However, considering that transaction costs constitute between 5–10% of the total costs in our pilot projects, potential value of investments to account for soil carbon would most likely outweigh the additional transaction costs. This could significantly reduce the REDD+ cost on a per tCO_2_ basis.

### Implementation costs

The implementation costs of the three pilot projects range between US$ 3.7 - 7.9 ha/year (US$ 4.5 - 12.2 tCO_2_) over a period of 30 years. Comparing with other global REDD+ implementation cost studies, Nepstad et al. [27] estimated implementation costs to US$ 0.58 tCO_2_ in the Amazon region based on historical data. Grieg-Gran [28] estimated administrative costs of REDD+ schemes on a national scale based on experience from nine REDD+ countries between US$ 4 and 9 per ha/year equivalent to US$ 0.01 - 0.02 tCO_2_. However, these costs only considered the management of PES schemes and did not take into account additional investments in changing land use management. Thus, direct comparisons cannot be made and provide only a highly uncertain indication of actual REDD+ implementation costs. As an example from Tanzania, implementation of activities for agricultural intensification, improved charcoal production and improved energy efficiency were identified as the major strategies to avoid emissions from deforestation and forest degradation [14]. The estimated implementation costs ranged between US$ 2.14 – 19.77 tCO_2_, with opportunity costs at US$ 1.90 – 13.40 tCO_2_. These estimates can be directly compared with data from the three REDD+ pilot projects described in this paper, that have implementation costs of US$ 4.5 - 12.2 tCO_2_ with mean project opportunity costs of US$ 10.1 – 12.5 tCO_2_. Our results indicate a close relationship between the level of opportunity cost and implementation cost that should be estimated in economic analyses of REDD+ to inform decision-makers of the potential cost-effectiveness and scale of REDD+ investments. Higher opportunity costs tend to have correspondingly higher implementation costs, which can be explained by the higher level of investment requirements to offset opportunity costs. Implementation of activities that focus on addressing the drivers of deforestation and forest degradation by increasing the profitability of one particular area of land, such as sustainable intensification of agriculture, may lead to the compensation of opportunity costs of land users with limited access to land from improved, more efficient and profitable production on a per ha basis. However, on a landscape level the opportunity costs may rise as the land profitability from alternative land uses increases, and this may even increase the pressure for deforestation [29]. Therefore, implementation of REDD+ related activities that increase the profitability of land must be aligned with the design of targeted policies that stimulate non-farm employment in rural areas combined with effective land use planning and law enforcement in order to prevent further encroachment into forested areas [29].

### Transaction costs

In our estimates, REDD+ transaction costs for MRV and other carbon market related compliance costs ranged between US$ 0.2 and 0.9 ha/year which is equivalent to US$ 0.21 (Kilosa) - 1.46 tCO_2_ (JGP). Overall transaction costs are normally relatively scale invariant as project development entities normally have to invest into the establishment of a project-specific MRV system, undertake certification by internationally recognised standards and design and negotiate contracts [30]. Therefore, larger projects tend to have lower transaction costs on a per ha or per tCO_2_ basis due to the distribution of costs over a larger amount of project area and carbon benefits [6]. A study estimated transaction costs for Afforestation/Reforestation Clean Development Mechanism (A/R CDM) projects in a range of US$ 0.03 tCO_2_ for large projects to US$ 4.05 for smaller projects with a weighted average of US$ 0.26 tCO_2_ for all projects [6]. Another study estimated average transaction costs from eleven forestry carbon projects at US$ 0.36 tCO_2_ ranging between US$ 0.66 – 16.4 tCO_2_[31]. The World Bank Bio Carbon Fund experience lasting recent years of the development and implementation of A/R CDM projects in 16 countries indicates higher transaction costs that exceed US$ 1 per tCO_2_ ranging between 0.5% and 20% of projects’ total investments [32].

These cost estimates are in-line with our project-specific estimates and it can be concluded that REDD+ transaction costs tend to increase with the reduction of the project area and carbon benefits due to economies of scale [32-34]. Considering that REDD+ projects or programmes are normally very large, comprising several thousands of hectares, transaction costs are likely to constitute only a small portion of the total REDD+ costs, particularly if projects or programmes are implemented at regional or national scales. In addition, the greater the scale of emissions reductions on a specific area, the lower the cost per tCO_2_[22].

### Institutional costs

In previous studies, institutional costs were not explicitly estimated or described. Administrative and implementation costs are likely to comprise institutional costs. A strict differentiation between institutional costs and implementation costs is very difficult to achieve, since in some stages costs could be assigned either to implementation or institutional costs. Moreover, national expenditures for forest management may reflect the business as usual scenario and are not additional REDD+ investments, and thus cannot be reliably accounted for. Therefore, the biggest challenge to estimating institutional costs is the determination of incremental costs related to the design and implementation of a REDD+ scheme. Institutional costs of REDD+ are defined in the context of this study as costs incurred at the political-administrative level to develop, manage and enforce REDD+ policies and measures. These are typically costs incurred by the government to ensure a positive legal and regulatory environment, address governance issues and reduce unregulated / illegal forest use. Our institutional cost estimates on the national level of US$ 0.017 ha/year provides an initial benchmark on institutional REDD+ costs at national level. However, since Tanzania is still in the development of the REDD+ implementation framework it is likely that additional institutional costs will arise compared to those currently budgeted. Therefore our estimates tend to underestimate the actual institutional costs which will need to be further updated once the REDD+ implementation framework is operational.

## Conclusions

Global and national REDD+ cost assessments have mostly been conducted in moist tropics with high forest density and using highly aggregated data in REDD+ countries such as Brazil, Indonesia, Democratic Republic of Congo, Ghana, and Cameroon. These aggregated estimates do not reflect the economic conditions, regional variations and forest carbon densities of dry forest ecosystems such as are prevalent in Tanzania, and tend to underestimate the actual costs of REDD+ in these regions. The focus on opportunity costs of REDD+ is only one crucial part of REDD+ economics providing information on the economic baseline situation of deforestation and forest degradation agents and on the required economic incentives to halt unsustainable land use. However, since performance-based REDD+ payments are expected to decrease over time (as deforestation rates drop and because payments for each saved ha of forest occur only once), these agents will require new revenue streams that compensate their opportunity costs and help to maintain natural forests in the long-term. Therefore investments into implementation of REDD+ activities comprising about 89 - 95% of total costs (excluding opportunity costs), must result in a more sustainable land use systems that the returns exceed the opportunity costs. Thus investments in REDD+ implementation paid for by decreasing performance-based carbon payments have a crucial role in triggering sustainable land use systems by investing in the underlying assets of the project. Focusing only on carbon payments bears the risk of temporally shifting unsustainable land use without addressing the underlying causes of deforestation and forest degradation. With the increased profitability of land use due to investments in effective REDD+ implementation, investments in institutional costs must create an enabling environment for REDD+ and policies that do not impose more pressure on natural forest, e.g. through increased profits from agricultural intensification.

Compared to top-down REDD+ cost assessments, bottom-up approaches to estimating REDD+ economics by considering regional variations in economic conditions is a more appropriate approach to provide policy and decision-makers robust economic information on which REDD+ decisions, policies, strategies and investments should be based on. Estimating the full range of REDD+ costs, including opportunity costs, implementation costs, transaction and institutional costs, must be complemented by the development and estimation of potential revenues to assess the potential effectiveness of REDD+ investments. This will provide data for the design of marginal abatement cost curves (MACC) as a robust economic decision-making tool which easily communicates information to multiple stakeholders on the mitigation potential and the incremental cost of different mitigation practices [35]. Beyond this, economic analysis should be accompanied by an assessment of non-market ecosystem services of forest such as biodiversity, water, soil conservation and social and environmental impacts in order to develop and implement efficient, equitable and effective REDD+ with multiple benefits.

## Methods

### Study sites

As part of the Tanzanian national REDD+ strategy, nine REDD+ pilot projects [36] have been initiated since 2009 with the objective of informing the development of the national REDD+ framework and building local capacity to implement REDD+ [37]. Due to time and resource constraints we selected the three most advanced of the nine REDD+ pilot projects to assess the cost elements of REDD+. The total area covered by these three projects was 327,825 ha (Table 1). Criteria for the selection were that the projects were close to their implementation stage, and that data on costs and land use change patterns were available. In addition, regional representativeness among different Tanzanian regions and different drivers of deforestation, forest degradation and ecosystems were used as supplementary selection criteria. The selection process was conducted during a REDD+ workshop on the 5^th^ of October 2011 in Dar es Salaam (Tanzania).

### Data collection

Data collection was conducted through project visits and the collection of project related available information. Project information included socio-economic studies, analyses of the drivers of deforestation, documented project-specific carbon assessments, project funding proposals and existing project budgets and expenditure data. Missing information required for a complete REDD+ cost assessment was complemented by local expert estimates for the respective pilot project. Expert estimates made by the authors were verified by the local project staff members.

### REDD+ cost element definitions

Since cost definitions often vary among different REDD+ cost assessments, we defined the four REDD+ cost elements following Pagiola et al. and White et al. [22,38].

### Opportunity costs

Opportunity costs are often the largest element of REDD+ costs. These equate to the costs of foregone benefits (such as producing crops and other agricultural activities) incurred by retaining existing forest land that could have been otherwise used for the economic activities. REDD+ opportunity costs are the difference in net earnings from conserving or enhancing forests and the earnings from converting them to alternative land uses. These costs are compared with potential benefits of maintaining the forests and carbon, for instance, by conserving the forest or extracting non-timber forest products. Opportunity costs can be quantified in terms of monetary and physical units, such as US$ per tCO_2_. Opportunity costs are profits achieved by continuing “business as usual” and depend on the drivers of deforestation and forest degradation. Estimating these costs is also critical in order to understand the causes and the motivations of deforestation agents to deforest, and are a part of each REDD+ pilot activity nationally and sub-nationally.

### Implementation costs

Implementation costs are defined as the costs and investments required to implement REDD+ activities and to avoid and minimize leakage effects. This includes, for instance the costs of guarding a forest to prevent illegal logging, costs of intensifying agriculture or pasture management, or improvement of energy efficiency so that people are not forced to collect fuel wood and produce charcoal for subsistence uses. Implementation costs also include capacity building of the project team to enable them to effectively manage and implement projects, costs of land use planning and other related activities required to achieve reduce emissions from deforestation and forest degradation.

### Transaction costs

Transaction costs are expenditures that are required to realise a transaction involving a REDD+ payment (buyer and seller, or donor and recipient) as well as external parties such as market regulators or payment scheme administrators [22]. REDD+ transaction costs are related to the MRV of forest carbon, other carbon market compliance costs, marketing of GHG benefits and registry operations that track and administer the carbon asset. These costs are normally borne by project or programme implementing entities. These costs differ from implementation costs in that they do not specifically reduce emissions from deforestation and forest degradation but are crucial to access potential performance based REDD+ payments.

### Institutional costs

Institutional costs are costs related to the implementation of conventions, norms and legal rules of a REDD+ framework so as to make REDD+ operational, including the costs of building institutions (and their capacity), strengthening land tenure, building an enabling policy environment. This includes expenditures associated with training, research, policy design, legal and regulatory processes, law enforcement as well as national stakeholder consultations and decision making costs. These costs may be very difficult to estimate but have to be taken into account [5,22]. For this cost type it is important to differentiate between business-as-usual and additional REDD+ related institutional investments. In our study we defined REDD+ institutional costs as costs that are additional to the historic forestry related national government budgets.

### Data assessment

Conceptually, this study estimates the REDD+ cost elements based on a project specific empirical approach using project-specific per-area estimates (US$/ha) and carbon density estimates (tCO_2_/ha). For each pilot project, we developed a standardized “REDD+ cost elements” analytical excel-based tool to separately estimate the opportunity costs, implementation, transaction and institutional costs. The tool is based on the methodological approach described in “Estimating the Opportunity Costs of REDD+ − A training manual” [38]. Moreover, the tool was developed with a focus to integrate the generated data into the REDD+ Abacus visualization tool that enables generation of opportunity cost curves for REDD+ projects and regions [39].

### Opportunity costs

Estimation of opportunity costs was conducted in four iterative steps, including classification of current land use types in the project area (ha) (1), an analysis of historical spatial land use change patterns and trajectories (ha) (2), estimation of mean carbon stocks of the identified land use types (tCO_2_/ha) (3) and estimation of one ha economic models of the land uses (NPV in US$/ha) (4). Quantitative data from these steps was input into the Abacus software to generate opportunity cost curves [39]. The calculation of opportunity costs was done using the following formula:

(1) $$OC_{\mathrm{nat}}=\left[\frac{NPV_{\mathrm{nat}}-NPV_{x}}{C_{x}-C_{\mathrm{nat}}}\right]$$

Where OC_nat_ is the opportunity costs of avoiding the conversion of one ha of natural forest to an alternative land use, expressed in US$/tCO_2_. NPV_nat_ are the discounted cash flows from natural forest, while NPV_x_ are the discounted cash flows related to a particular driver of deforestation or forest degradation, both discounted by 10% over a period of 30 years, expressed in US$/ha. Cx is the mean carbon stock value (aboveground and belowground) of a particular driver of deforestation and forest degradation, while Cnat is the mean carbon stock of of natural forest, expressed in tCO_2_/ha. The calculation of opportunity costs was conducted only for land that has been converted from natural forest to alternative land uses over the past ten years, as described in the results section (REDD+ opportunity costs curve). The mean opportunity costs of each project were calculated by weighting the relative contribution of each driver of deforestation and forest degradation to the total forest loss in the project area.

### Classification of land use types and carbon stocks

In the classification of land use types, each ha of the total project area must be assigned to a land use associated with a particular economic activity. In the three analyzed REDD+ pilot project, land uses such as natural forest, unsustainable charcoal production, unsustainable fuel wood collection, unsustainable timber extraction, agricultural (shifting cultivation) land uses and pastoral cattle grazing were identified. Unsustainable land uses are characterized by extraction rates that would not have been able to sustain the prevalent practices over 30 years, thus leading to deforestation and forest degradation.

Each identified land use type is assigned a long-term mean carbon stock value (in tCO_2_/ha). The long-term mean carbon stock reflects the equilibrium carbon stock of a particular land use. If land use is converted to another land use, we assume that carbon is emitted or sequestered immediately. In our study we considered forest carbon only from aboveground and belowground carbon pools, due to the fact that the projects are not planning to measure other carbon pools and receive payments related to these pools. Where natural forest land was comprised of more than one forest type with differing carbon stocks, we used area-weighted mean carbon stocks in which the carbon stocks of each forest type were multiplied by the area of the respective forest type and subsequently divided by the total natural forest area.

### Historical land use change patterns and GHG emissions

This step included a quantitative estimation of the project specific conversion of forest land to other identified land uses associated with a particular economic activity that has led to deforestation and forest degradation (in ha) (Table 5). In our assessment, land use changes were assessed based on the past ten years prior to the project start. This activity data forms the basis to quantify the carbon dioxide emitted over this reference period for each driver of deforestation and degradation using the following formula:

(2) $$GHG_{\mathrm{em}}={\left(C_{\mathrm{nat}}-C_{x}\right)}^{*}A_{x}$$

**Table 5:** Project specific land use changes from 2001 – 2011

**Land use classification of REDD+ pilot projects**	**Jane Goodall Masito Ugalla ecosystem pilot area**	**Tanzania forest Conservation Group (TFCG) / Mjumita project - Kilosa site**	**Tanzania forest Conservation Group (TFCG) / Mjumita project – Lindi site**
**Natural forest**	Forest area loss from 85,200 ha to 71,037 ha	Forest area loss from 148,825 ha to 139,617 ha	Forest area loss from 93,800 ha to 76,992 ha
**Unsustainable timber extraction**	Conversion of natural forest from 0 ha to 3,000 ha		
**Unsustainable charcoal production**		Conversion of natural forest from 0 ha to 4,236 ha	Conversion of natural forest from 0 ha to 8,404 ha
**Unsustainable fuel wood production**	Conversion of natural forest from 0 ha to 4,000 ha		
**Agriculture (Shifting cultivation)**	Conversion of natural forest from 0 ha to 5,163 ha	Conversion of natural forest from 0 ha to 4,972 ha	Conversion of natural forest from 0 ha to 8,404 ha
**Pasture (Cattle grazing)**	Conversion of natural forest from 0 ha to 2,000 ha		

Where GHG_em_ are the total GHG emissions from conversion of natural forest to a particular land use (Table 5), expressed in tCO_2_. C_nat_ is the mean carbon stock value of natural forest in tCO_2_/ha, whereas C_x_ is the mean carbon stock value of land use associated with a particular driver of deforestation and forest degradation in tCO_2_/ha. A_x_ is the area converted from natural forest to a particular land use, expressed in ha over a period of 10 years.

### Economics of land use

The most sophisticated approach to estimate opportunity costs is to develop models of production and returns based on production inputs, yield and actual commodity prices in the respective regions [22]. In our analysis, we used this approach by developing project specific representative land use models for one ha for natural forest and each identified alternative land use (Table 5). We considered regional differences and local socio-economic and market conditions in our modelling work. For each ha we used the annual average inputs, costs, yields, annual average annual extraction rates, growth rates and revenues at farm-gate. We also valued in-kind family labour as an input cost and household level consumption assuming farm-gate prices which do not result in cash revenues, but have economic values to the land users that could have been sold to local markets. We included these costs since markets in rural areas of Tanzania often are not formalised, and excluding unpaid household labour and consumption would not reflect the actual value of land to the land users.

For the economic analysis we used the net present value (NPV) of net revenue streams associated with each land use as an economic indicator to estimate the profitability of land use. The NPV is the result of a Discounted Cash Flow analysis (DCF) of the costs and benefits for a certain land use over period of 30 years using a discount rate of 10%. Since our estimates are based on a period of 30 years and unsustainable timber extraction, charcoal production and fuel wood collection cannot be sustained over such a long time period, our model allowed a transition to another land use such as agriculture or pastoral use depending on local circumstances after the wood resource base would have been depleted, and this net revenues from this subsequent land use also contributed to the estimated NPV.

### Implementation and transaction costs

For the estimation of implementation and transaction costs we used existing REDD+ project documents that included budgets and expenditures over a period of three to five years and that were extrapolated over the project lifetime of 30 years using expert judgement. For the identification of this comprehensive set of cost elements and for the verification of expert judgement, we worked in close cooperation with project development and implementation entities. The assignment of each budgeted or already spent money to the respective cost type was based on the REDD+ cost element definition as elaborated in the background section. Due to the lack of data and existence of solid revenue models, in the project scenarios we did not take into account potential non-REDD+ revenue streams. Therefore we assume that all costs are covered by potential carbon payments and estimate the required carbon price to cover these costs.

### Institutional costs

For the determination of institutional costs we took into account only incremental REDD+ costs taken from the Tanzania’s REDD+ readiness and implementation plan prepared under the framework of the World Bank Forest Carbon Partnership Facility (FCPF) and UN-REDD programme budgets between 2010 and 2013, which amounted to US$ 3.47 million [18]. Subsequently we extrapolated this budget to the project lifetime of 30 years by dividing this budget by the total national forest area of 35.3 million ha, resulting in an annual average value of US$ 0.17 ha/yr. We used this institutional cost (US$/ha) value and multiplied it by the total project area which we included in the project specific institutional cost estimates.

We did not add up the existing forestry related budget and expenditures of governmental institutions, as we consider these business-as-usual that would have occurred without a REDD+ framework anyway. Therefore our estimates include only REDD+ specific “additional” costs. In the framework of this study, “additional” costs are considered as expenditures being spent in the framework of national REDD+ Readiness and Implementation activities financed by the UN-REDD Programme, FCPF, Norway-Tanzania Climate Change Partnership and by the government of Tanzania [18].

Furthermore for all projects, we assigned a small portion of the budget to local-level institutional capacity building and REDD+ knowledge dissemination, which we considered as an institutional cost according to the definition elaborated in the background section. These costs will be paid from project budgets. Finally we summed the national level and project level institutional costs.

### Calculation of costs per ha and per avoided GHG emission (tCO_2_)

For the estimates of REDD+ cost elements on a per ha basis (US$/ha) we divided the annual average implementation, transaction and institutional costs of the pilot projects by the total project area. For the estimates of the costs per avoided tCO_2_ (US$/tCO_2_) we used the projected GHG emissions reductions from the avoidance of deforestation and forest degradation. Based on these estimates we divided the total implementation, transaction and institutional costs over a period of 30 years by the total projected GHG emissions reductions in the project scenario.

### Uncertainties and limitations

REDD+ cost curves and estimates reflect only a static snapshot of one period of time and the current economic and political conditions prevalent in the project regions, and do not reflect potential future developments [35]. Particularly opportunity costs may be subject to significant changes due to population growth, political or economic changes, thus the information provided by opportunity cost curves can only inform decision-makers on the required level of REDD+ related revenues streams under the current political and economic conditions. The limitation of this cost estimation approach is that potential intersectoral economic changes that may have significant effects on opportunity costs and/or implementation costs to address the drivers of deforestation are not accounted for. Therefore, for decision-makers it is important to take into account interactions between the adoption of REDD+ and other sectoral strategies and policies [40]. The major benefits of REDD+ cost information is the support to decision-making in the allocation and prioritization of limited REDD+ funds to activities and investments, and the utility of this analysis in assessing how much benefit may be derived from alternative and total investments [1].

Another uncertainty of the REDD+ cost estimate lies in the future projection of project investments and estimates of the future GHG emissions reductions that will be actually achieved. It remains unclear whether the budgeted investments will actually result in the expected carbon savings, thus the effectiveness of REDD+ project investments still has to be proven by the successful implementation of the projects, supported by an enabling environment of the national REDD+ framework.

## Competing interests

The authors declare that they have no competing interests.

## Authors’ contributions

EM designed the analytical REDD+ cost element tool, undertook the pilot project analysis, conducted interviews with local experts of the respective pilot project together with CH and drafted the manuscript. CH co-authored the manuscript. CH was mainly responsible for data collection, communication with the project development and implementation entities and quality control. TT and TB were responsible for review of the manuscript, provision of comments and quality control. All authors read and approved the final manuscript.

## Authors’ information

EM works as a land-based climate finance project developer and analyst at UNIQUE forestry and land use. CH is Managing Partner of UNIQUE forestry and land use, heading the Forest Economics Division. TTe is Managing Director of UNIQUE forestry and land use and head of the Climate Division. TB is a freelance consultant regularly working for LTS International (UK). From 2003 to 2008 he worked as senior participatory forest management adviser for the Government of Tanzania within the Forest and Beekeeping Division.
